# Asymmetrically twisted phenanthrimidazole derivatives as host materials for blue fluorescent, green and red phosphorescent OLEDs

**DOI:** 10.1038/s41598-019-54125-x

**Published:** 2019-11-26

**Authors:** Jayaraman Jayabharathi, Sekar Panimozhi, Venugopal Thanikachalam

**Affiliations:** Department of Chemistry, Annamalai University, Annamalai nagar, 608 002 Tamilnadu India

**Keywords:** Chemistry, Materials chemistry, Optical materials

## Abstract

The electroluminescent properties of asymmetrically twisted phenanthrimidazole derivatives comprised of fluorescent anthracene or pyrene unit namely, 1-(1-(anthracen-10-yl)naphthalen-4-yl)-2-styryl-1H-phenanthro[9,10-d]imidazole (ANSPI), 1-(1-(pyren-1-yl) naphthalene-4-yl)-2-styryl-1H-phenanthro[9,10-d]imidazole (PNSPI), 4-(2-(4-(anthracen-9-yl) styryl)-1H-phenanthro[9,10-d]imidazol-1-yl)naphthalene-1-carbonitrile (ASPINC) and 4-(2-(4-(pyren-1-yl)styryl)-1H-phenanthro[9,10-d]imidazol-1-yl)naphthalene-1-carbonitrile (PSPINC) for blue OLEDs have been analyzed. The asymmetrically twisted conformation interrupt π-conjugation effectively results in deep-blue emission. The pyrene containing PSPINC based non-doped blue device (476 nm) shows maximium efficiencies (current efficiency (*η*_*c*_)-4.23 cd/A; power efficiency (*η*_*p*_)-2.86 lm/W; external quantum efficiency (*η*_*ex*)_-3.48%: CIE (0.16, 0.17) at 3.10 V. Among the doped blue devices, An(PPI)_2_:ASPINC shows high efficiencies (*η*_*c*_-12.13 cd/A; *η*_*p*_-5.98 lm/W; *η*_*ex*_-6.79%; L-23986 cd m^−2^; EL-458 nm) at 3.15 V with CIE (0.15, 0.17) than An(PPI)_2_:PSPINC based device which is inconsistent with non-doped device performances. The green and red PhOLEDs show higher efficiencies with Ir(ppy)_3_: ASPINC (*η*_*c*_-50.6 cd/A; *η*_*p*_-53.4 lm/W; *η*_*ex*_-17.0%; L-61581 cd m^−2^; EL-501 nm, CIE (0.31, 0.60) at 3.32 V and (bt)_2_Ir(dipba): ASPINC (*η*_*c*_-15.2 cd/A; *η*_*p*_-16.5 lm/W; *η*_*ex*_-14.5%; L-13456 cd m^−2^; EL-610 nm), CIE (0.63, 0.36) at 3.20 V, respectively. The complete energy transfer between the host and dopant molecules improved the efficiency of PHOLEDs.

## Introduction

Blue electroluminescent devices remain the bottleneck for high chromaticity and short lifetime relative to green or red OLEDs^1–5^. Phenanthroimidazole derivatives (PPI) have been widely used as a building block for blue OLEDs because of effective bipolar-materials with high stability: non-doped blue device with 4,4′-bis(1-phenyl-1H-phenanthro[9,10-d]phenanthroimidazolyl-2-yl)biphenyl (PPIP) emissive material shows high *η*_*ex*_ and *η*_*p*_ of 6.31% and 7.30 lmW^−1^, respectively^6^. The blue device (CIE-0.15, 0.21) with PPIP based bifunctional electroluminescent (EL) material exhibit high *η*_*c*_ of 6.87 cd A^−1^at 2.8 V^7^. Blue PPI with D-π-A configuration show *η*_*ex*_ of 7.8% and *η*_*c*_ of 10.4 cdA^−1^ ^8^, device efficiencies of blue materials suffer from carrier injection and transportation in the emissive layer^9^; blue devices with anthracene derivatives have been widely attracted due to unique EL properties^10^; however, easy crystallization in film state limits their applications.

The bulky substituent at C-9 and C-10 of anthracene derivatives show blue emission due to rupturing of π-conjugation because of non-coplanar geometry leads^10,11^. This asymmetric architecture minimized the crystallization and enhances the amorphous morphological stability leads to enhanced efficiency^11–13^. The 3,6-di(anthracen-9-yl)-9-phenyl-9H-carbazole and fluorine based naphthyl anthracene derivatives with reduced close-packing exhibit high *η*_*c*_ of 3.14 cd A^−1^ with CIE (0.16, 0.14) at 3.8 V and 4.04 cd A^−1^ with CIE (0.15, 0.13) at 4.1 V, respectively due to high fluorescent quantum yield^14,15^. We have designed some asymmetric PPI derivatives substituted with anthracene group^16^, which can increase the electron injection/transportation to reduce hole injection barrier (HIB), results in high efficiency. The green and red PhOLEDs with 100% IQE (internal quantum efficiency) have been reported^17–20^, but efficient blue PhOLED have not yet been achieved: poor blue colour purity (CIE ≥ 0.20) with poor EL efficiency have been reported^21–25^. Also energy loss (≈0.5–1.0 eV) due to energy transfer (host to dopant) process leads to poor efficiency^26^. Hence, development of efficient blue OLEDs from stable blue fluorescent emitters in flat-panel display is an urgent need in OLEDs ^27–31^. The primary emitters urgently need special hosts for efficient energy transfer^31–38^.Therefore, development of full-color blue FLOEDs, RG PHOLEDs and white OLEDs *via* simple material is an important issue for OLED applications. Although, many multi-color and white OLEDs with universal host have been reported^6,39–41^, synthesis of a host for efficient blue fluorescent OLEDs as well as phosphorescent green and red OLEDs is still a considerable challenge. Highly stable phenanthrimidazole derivatives with high electron injection/transporting ability are widely used as EL materials^6,41–47^, however, efficiency roll off is the major problem^48–51^. Thus, coupling of anthracene or pyrene moieties at nitrogen and carbon of phenanthrimidazole play a vital role in designing blue emissive materials with minimized efficiency roll-off^52–58^. With this aim, we have designed asymmetrically twisted phenanthrimidazole derivatives coupled with anthracene**/**pyrene units such as ANSPI, PNSPI, ASPINC and PSPINC exhibit high-energy emission and as also employed a high-performance host material due to good carrier-transport ability.

## Results and Discussion

**Scheme 1:**
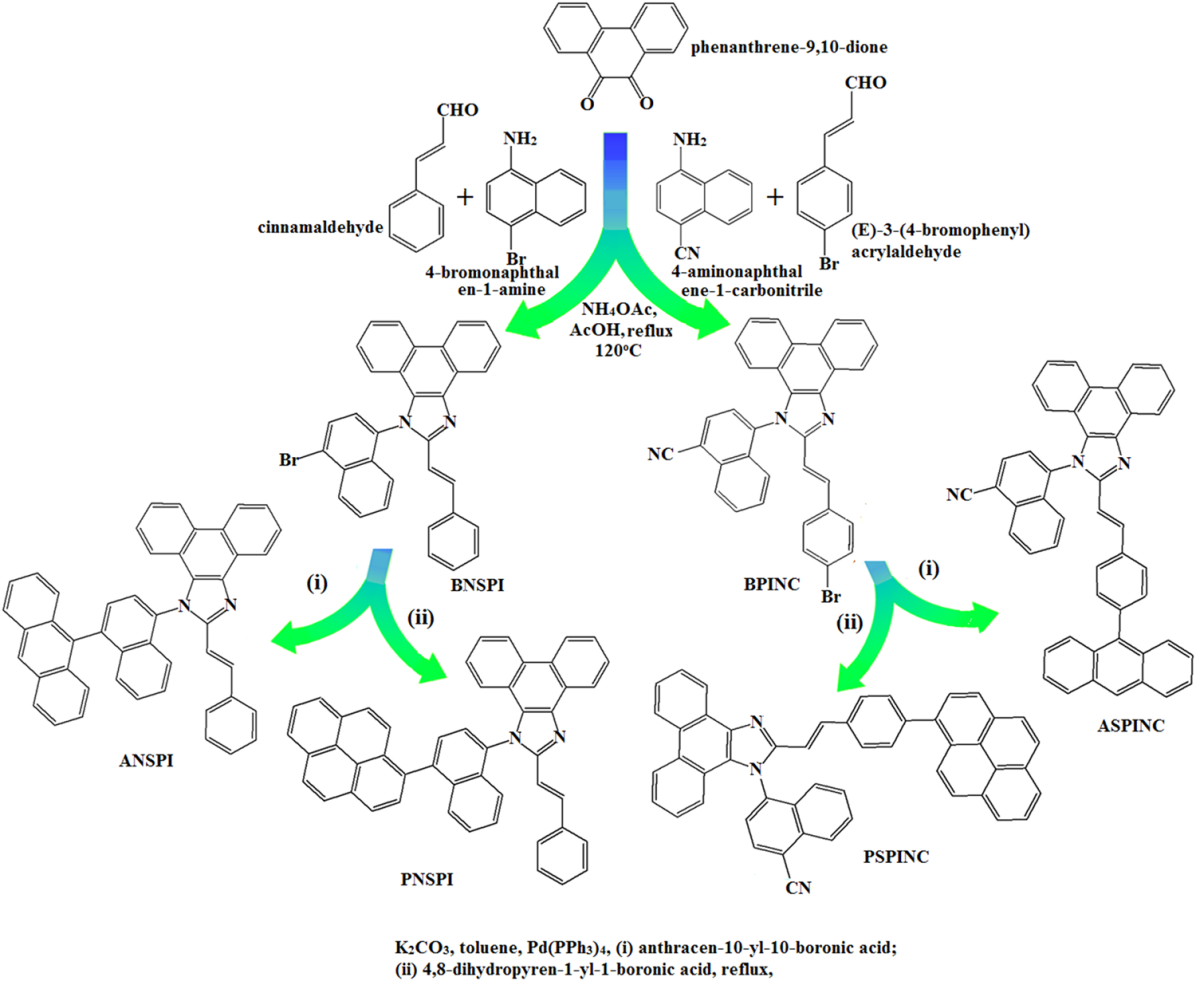
Synthetic route of ANSPI, PNSPI, ASPINC and PSPINC.

The synthesis of efficient blue emitters as well as host materials namely, 1-(1-(anthracen-10-yl)naphthalen-4-yl)-2-styryl-1H-phenanthro[9,10-d]imidazole (ANSPI), 1-(1-(pyren-1-yl)naphthalen-4-yl)-2-styryl-1H-phenanthro[9,10-d]imidazole (PNSPI), 4-(2-(4-(anthracen-9-yl)styryl)-1H-phenanthro[9,10-d]imidazol-1-yl)naphthalene-1-carbonitrile (ASPINC) and 4-(2-(4-(pyren-1-yl)styryl)-1H-phenanthro[9,10-d] imidazol-1-yl) naphthalene -1-carbonitrile (PSPINC) were synthesized with appreciable yield of 59, 62, 60 and 64% through palladium-catalyzed Suzuki coupling reaction of the intermediates namely, 1-(1-bromonaphthalen-4-yl)-2-styryl-1H-phenanthro[9,10-d]imidazole (BNSPI) and 4-(2-(4-bromostyryl)-1H-phenanthro[9,10-d]imidazol-1-yl)naphthalene-1-carbonitrile (BPINC) with corresponding aryl boronic acids (Scheme 1)^59^. The blue emitters ANSPI, PNSPI, ASPINC and PSPINC were purified by column chromatography and characterized by ^1^H, ^13^C NMR, mass and elemental analysis.

### Potential energy surface (PES) scan and thermal properties

**Figure 1:**
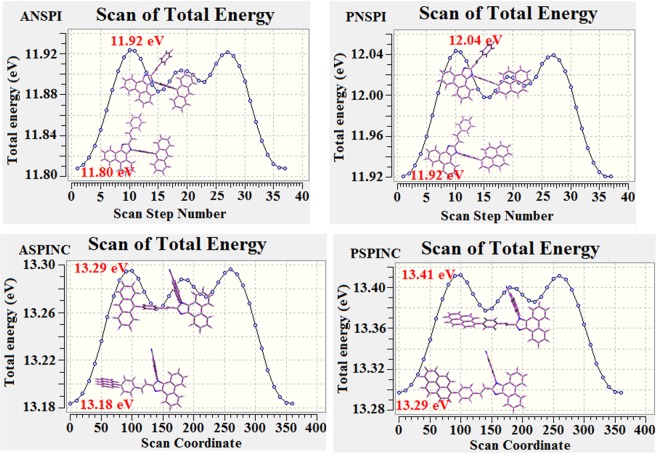
Potential energy scan of ANSPI, PNSPI, ASPINC and PSPINC.

**Figure 2:**
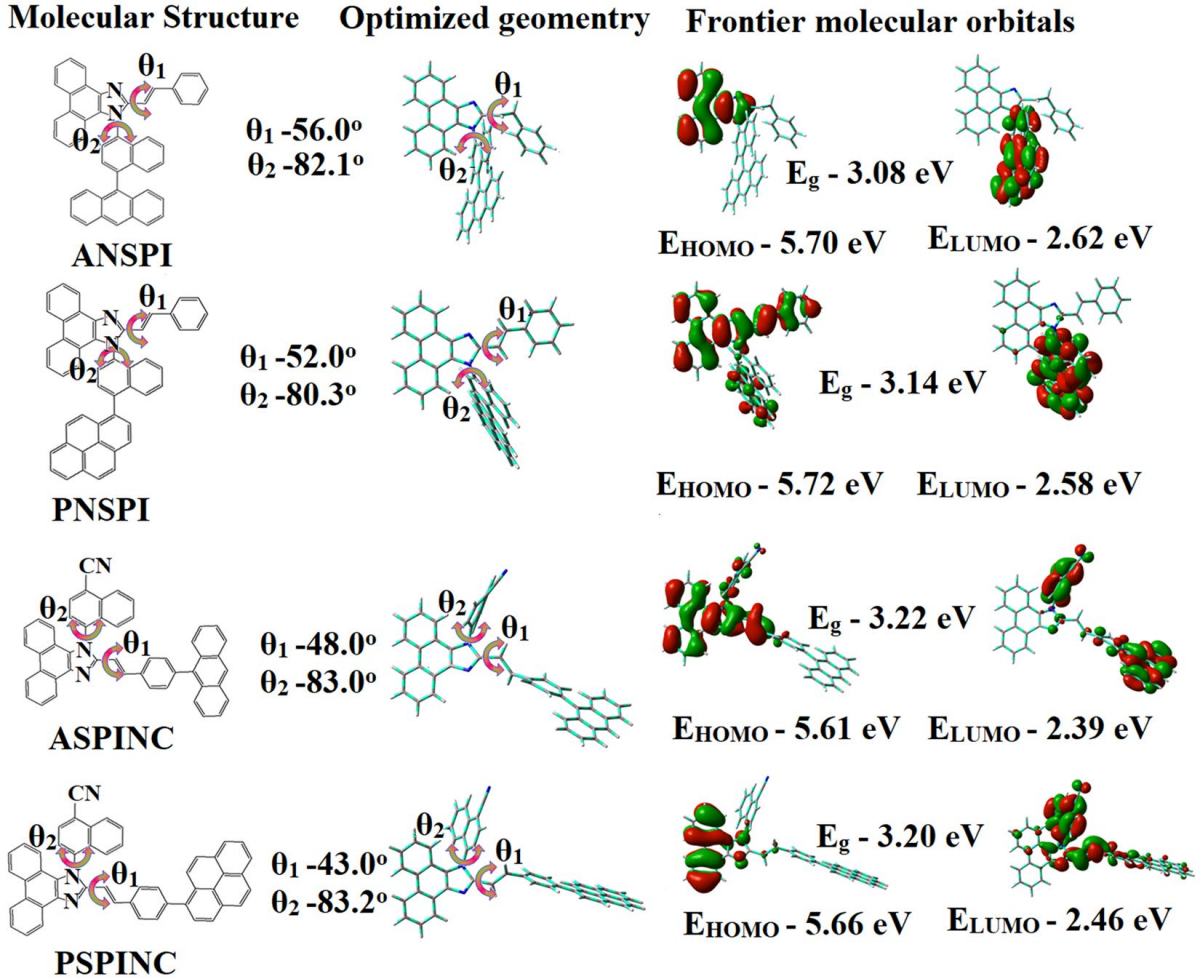
Molecular structure, ground state geometries with dihedral angles and Frontier molecular orbitals of ANSPI, PNSPI, ASPINC and PSPINC.

The configuration effect of blue emissive materials ANSPI, PNSPI, ASPINC and PSPINC on optical properties have been analyzed theoretically (DFT/B3LYP/6–31 G (d)) (Fig. 1). The potential energy surface scan about C24-C25-C26-C27 (ANSPI, PNSPI, ASPINC and PSPINC) have been performed. The minimum energy conformation of ANSPI, PNSPI, ASPINC and PSPINC shows that the orthogonal dihedral angle (~83.0°) between the side chain at N 23 and substituent at C 25 *i.e*., 10-(4-vinylphenyl)anthracene (ASPINC) and 1-(4-vinylphenyl)pyrene (PSPINC)**/**styryl (ANSPI, PNSPI) and side coupling of naphthyl anthracene (ANSPI)/1-naphthylpyrene (PNSPI)/naphthonitrile (ASPINC and PSPINC) at N 23 reduces the intermolecular packing. Therefore, the side chain at N 23 and rigid frame at C 25 used as hole-trapping sites and peripheral core blocks the electron-trapping sites. Hence, effective carrier injection and transport ability will be expected from these reported blue emitters (ANSPI, PNSPI, ASPINC and PSPINC). The ANSPI, PNSPI, ASPINC and PSPINC display highly twisted molecular conformation which is shown by the dihedral angle between the imidazole plane and bulky substituent at C25 (ANSPI-56°, PNSPI-52°, ASPINC-48° and PSPINC-43°) (Fig. 2). The incorporation of side capping at N 23 and substituent at C 25 enhanced the degree of molecular distortion and suppresses the aggregation formation/π-π stacking in film which results amorphous film during fabrication^60^. These orthogonal dihedral angles confirmed the non-coplanar twisting conformation of ANSPI, PNSPI, ASPINC and PSPINC results in high quantum efficiency in film by restraining intermolecular interaction^61–64^.

**Figure 3:**
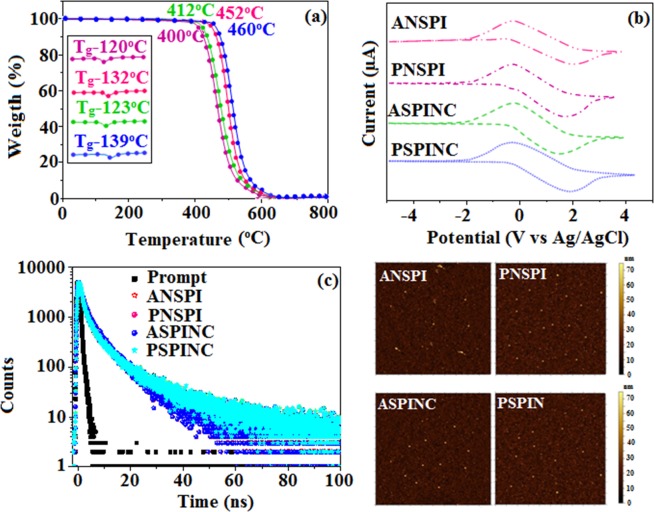
(**a**) TGA (inset: DSC) graph (**b**) Cyclic voltamogram (**c**) Lifetime spectra and (**d**) AFM images of ANSPI, PNSPI, ASPINC and PSPINC.

The phenanthrimidazole ring coupled with anthracene/pyrene moieties and styryl fragment at N 23 and C 25 position to form an asymmetrically twisted structure enhanced the thermal stability. The blue emissive materials exhibit maximum thermal stability as evidenced by decomposition temperature (*T*_*d*_) (corresponding to 5% weight loss): 400°-ANSPI, 452°-PNSPI, 412^°^-ASPINC and 460^°^C -PSPINC. This will prevent the decomposition of these materials during vacuum deposition and device operation processes. The high T_*d*_ indicates the high resistance of fused aromatic ring on thermolysis and the high T_*d*_ could enhance the device lifetime (Fig. 3)^65–69^. These materials has the ability to form an amorphous glass with a high glass-transition temperature (*T*_*g*_) of 120° -ANSPI°, 132° -PNSPI, 123° -ASPINC and 139 °C -PSPINC which is beneficial for the formation of stable, homogeneous and amorphous film upon thermal evaporation and decreases the phase separation of host–guest system when used as host material (Fig. 3: Table 1).

**Table 1:** Optical and thermal properties of ANSPI, PNSPI, ASPINC and PSPINC.

Emitters	ANSPI	PNSPI	ASPINC	PSPINC
λ_ab_(nm) (soln/film)	343,354,366/360	332,339,355/335,342	346,358,369/357,372	333,341,360/338,344
ε (M^−1^cm^−1^)	28248.59	29498.53	27932.96	29325.51
λ_em_(nm) (soln/film)	405,410/445	390,395/460	430/450	420/465
T_*g*_/T_*d5*_ (°C)	120/400	132/452	123/412	139/460
ɸ (soln) HOMO/LUMO (eV)	70–5.70/−2.62	60–5.72/−2.58	89–5.61/−2.39	76–5.66/−2.46
E_g_ (eV) τ (ns) k_r_ × 10^8^ (s^−1^) k_nr_ × 10^8^ (s^−1^)	3.08 3.9 1.7 0.7	3.14 4.3 1.3 0.9	3.22 4.6 1.7 0.4	3.20 4.5 1.5 0.6

The thermal morphological stability of ANSPI, PNSPI, ASPINC and PSPINC thin film were examined by AFM measurement (30° and 110 °C for 10 h). The RMS (root-mean-square roughness) of ANSPI (0.41 nm), PNSPI (0.46 nm), ASPINC (0.38 nm) and PSPINC (0.31 nm) thin-film surface reveal that there is no substantial changes before and after annealing (Fig. 3). The excellent thermal and amorphous stability indicates that phenanthrimidazole moiety comprised of anthracene and pyrene fragments may influence the arrangement of the molecules in the thin film and supports the suitability of these emissive materials for fabrication of blue OLEDs^65–67^.

### Electrochemical and photophysical properties

The onset oxidation potential (E_ox_) for ANSPI, PNSPI, ASPINC and PSPINC are 0.90, 0.92, 0.81 and 0.86 eV versus ferrocenium/ferrocene redox couple, respectively (Fig. 3). Thus, the HOMO energies were estimated to be −5.70, −5.72, −5.61 and −5.66 eV, respectively^70–72^, the E_HOMO_ of these materials is higher than that of fluorescent host material 4,4′-N,N′-dicarbazolylbiphenyl (CBP~ −6.0 eV) and matches well with widely used hole transporting material NPB implying that only little hole-injection barrier between NPB and these materials. The lower energy barrier between emitting layer, ANSPI, PNSPI, ASPINC and PSPINC and hole transporting layer will facilitate effective hole injection into emission layer.

The calculated LUMO energies [−2.62 eV -ANSPI; −2.58 eV- PNSPI; −2.39 eV-ASPINC; −2.46 eV-PSPINC] are in close with 1, 3, 5-tris(N-phenylimidazol-2-yl)benzene supports the electron injection abilities. The frontier molecular orbital analysis also confirms the carrier injection abilities and they can employed as potential emitters in OLEDs^71,72^. The optimized molecular geometry reveal that the π-conjugation between the phenanthrimidazole and anthracene (ANSPI & ASPINC)/pyrene (PNSPI & PSPINC) is interrupted that could induce the blue emission. The N 23-substituent is in a perpendicular direction which tends to inhibit π-π intermolecular interaction. The HOMO of ANSPI and PNSPI is mainly localized on naphthylanthracene and pyreneanthracene units at N 23, respectively and LUMO of ANSPI and PNSPI is located on phenanthrimidazole and styryl units. The HOMO of ASPINC and PSPINC is localized on anthracene/pyrene and phenyl of styryl fragment at C 25, respectively and LUMO is located on cyanonaphthyl at N 23 of phenanthrimidazole core. The significant spatial separation of HOMO and LUMO levels suggest that the HOMO–LUMO excitation would shift the electron density distribution from donor to acceptor of ANSPI, PNSPI, ASPINC and PSPINC leading to a polarized excited state. Such separation can provide hole- and electron-transporting channels where holes and electrons can realize intermolecular hopping smoothly along their respective conducting pathways. This indicates that ANSPI, PNSPI, ASPINC and PSPINC are bipolar materials with charge transport properties which is the requirement for host materials. The balanced carrier transport properties play a key role in conducting both holes and electrons and thus, improved the efficiency^73^.

**Figure 4:**
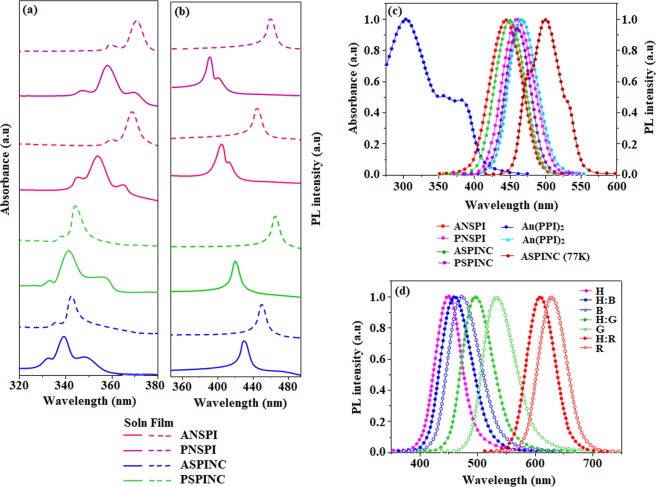
Normalized absorption (**a**) and emission (**b**) spectra of ANSPI, PNSPI, ASPINC, PSPINC; (**c**) UV-vis absorption spectra of An(PPI)_2_ and PL spectra of ANSPI, PNSPI, ASPINC, PSPINC and PL spectrum of ASPINC at 77 K; (**d**) PL spectra of neat film of ASPINC (H), blue fluorophore, green and red phosphors and these dopant emitters doped in ASPINC (H) thin film at 5 wt% doping concentration.

The UV-vis absorption and PL (low temperature and thin film) spectra of ANSPI, PNSPI, ASPINC and PSPINC in CH_2_Cl_2_ solution (10^−5^ mol L^−1^) were measured to evaluate their optical properties. A higher intensity absorption around 339 nm (354 nm-ANSPI, 339 nm-PNSPI, 358 nm-ASPINC and 341 nm-PSPINC) originates from π-π* transition of phenyl ring whereas the lower intensity absorption at 366 nm-ANSPI, 355 nm-PNSPI, 369 nm-ASPINC and 360 nm-PSPINC are attributed to π-π* transition of anthracene**/**pyrene units. The absorption spectra of vacuum-deposited thin film of these compounds are similar to the corresponding solution spectra in view of spectral profiles and wavelength. The PL spectra of ANSPI, PNSPI, ASPINC and PSPINC in CH_2_Cl_2_/film, ANSPI (410**/**445 nm), PNSPI (395**/**460 nm), ASPINC (430**/**450 nm) and PSPINC (420**/**465 nm) reveal the blue emission (Fig. 4). Compared with pyrenyl phenanthromidazoles (PNSPI and PSPINC), anthracenyl phenanthroimidazoles (ANSPI and ASPINC) show bathochromic shift due to conjugation^74^. The maximum emission of ANSPI, PNSPI, ASPINC and PSPINC in thin film is red-shifted compared to solution due to exciton hopping in film^75^, a gradual decreasing red-shift was observed with an increasing conjugation. It should be noted that the bulky substituent at N 23 and C 25 of phenanthrimidazole effectively limits the π-conjugation results in deep-blue emission.

The quantitative enhancement of emission was evaluated by quantum yield using quinine sulphate as a standard. These blue emitters ANSPI, PNSPI, ASPINC and PSPINC exhibit high quantum yield of 0.70, 0.60, 0.89 and 0.76, respectively; therefore, these compounds are excellent candidates for using as efficient emitting materials in OLEDs.

### Charge carrier injection and transport properties

**Figure 5:**
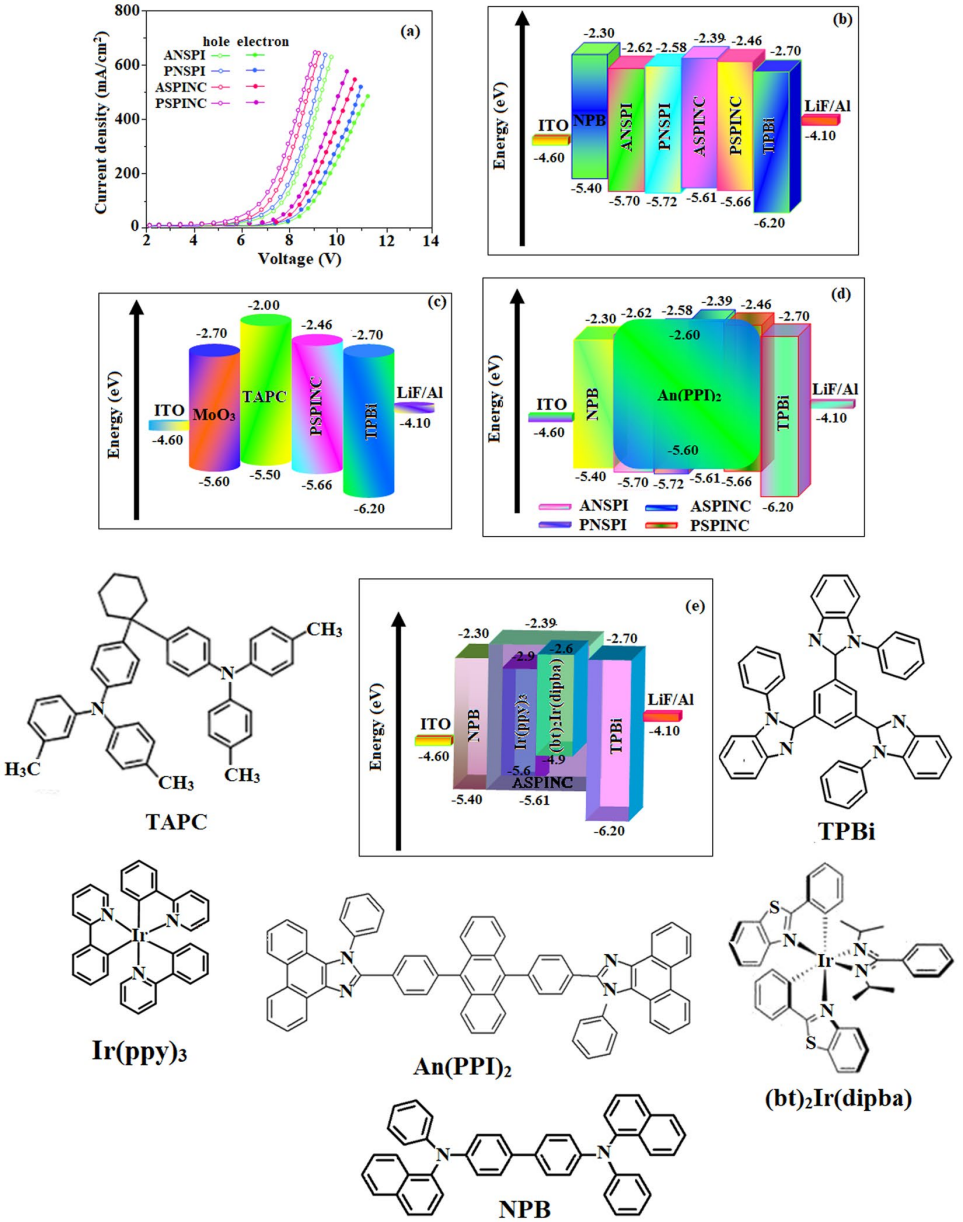
(**a**) Hole-only and electron-only devices; (**b**) Energy level diagram of non-doped blue device: ITO/NPB**/**ANSPI or PNSPI or ASPINC or PSPINC/TPBi/LiF/Al; (**c**) ITO/MoO_3_/TAPC/PSPINC/TPBi/LiF/Al; (**d**) doped blue device: ITO**/**NPB/ANSPI or PNSPI or ASPINC or PSPINC:An(PPI)_2_/TPBi/LiF**/**Al and (**e**) green and red devices: ITO**/**NPB**/**ASPINC: Ir(ppy)_3_ or (bt)_2_Ir(dipba)/TPBi/LiF/Al with molecular structures of functional materials.

To further understand the hole and electron injection/transport properties of ASPINC, single-carrier devices were fabricated: ITO/NPB (10 nm)/ANSPI/PNSPI/ASPINC/PSPINC (30 nm)/NPB (10 nm)/Al (100 nm) (hole-only device) and ITO/TPBi (10 nm)/ANSPI/PNSPI/ASPINC/PSPINC (30 nm)/TPBi (10 nm)/LiF (1 nm)/Al (100 nm) (electron-only device). The NPB and TPBi layers are employed to prevent electron and hole injection from cathode and anode, respectively^76,77^. These materials are efficient polar materials: capable of transporting both electrons and holes effectively leads to effective recombination of holes and electrons in the emitting layer (Fig. 5). However, the lower electron current than hole current at same voltage should be attributed to high-lying LUMO energy of emitters because of the injection barrier [−0.50 eV -ANSPI; −0.48 eV- PNSPI; −0.59 eV-ASPINC; −0.54 eV-PSPINC] from electron transport layer^78^.

### Electroluminescent performances

**Figure 6:**
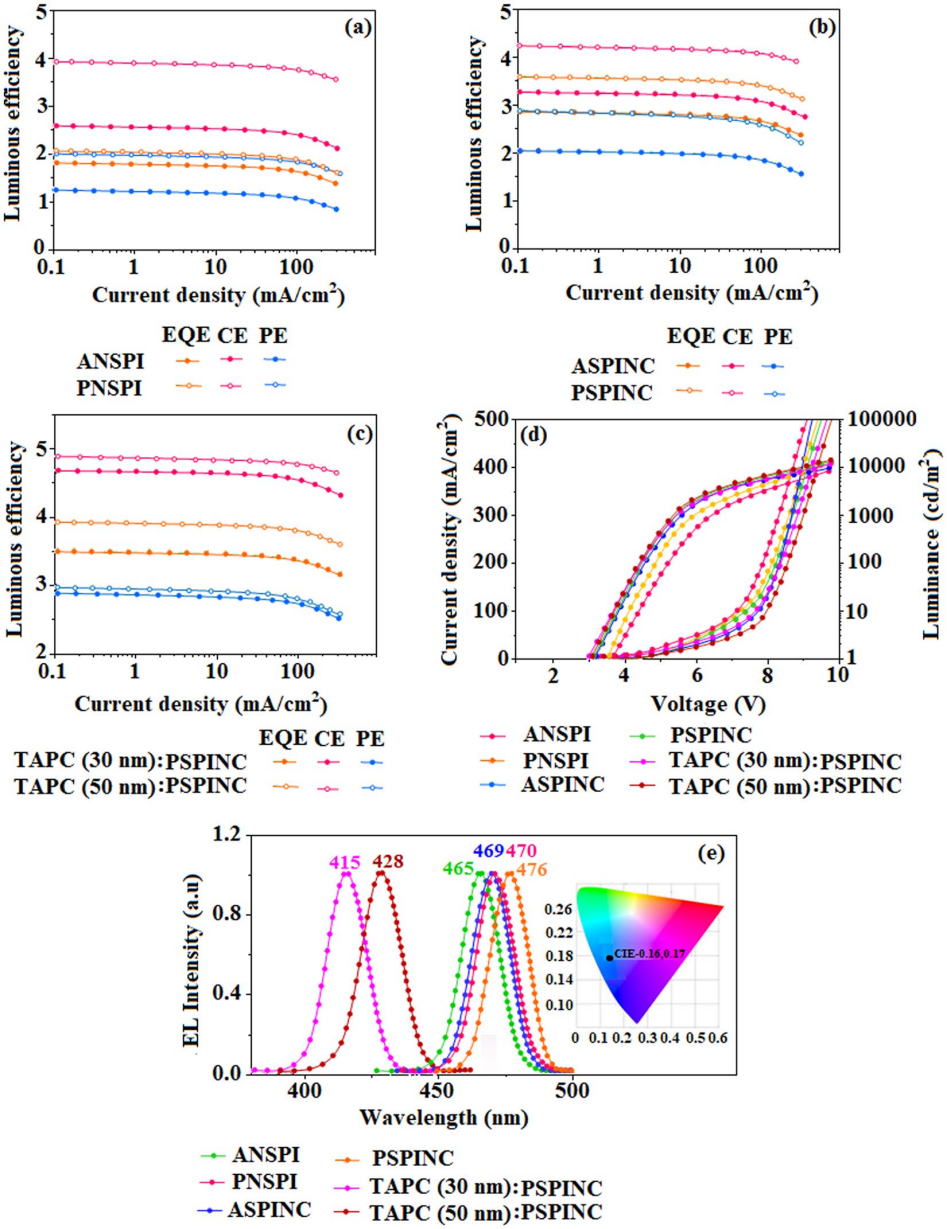
Device efficiencies of non-doped blue device: Luminous efficiency [CE (cd/m^2^), PE (lm/W)& EQE (%)] -Current density (**a**–**c**); Luminance –Voltage (**d**,**e**) EL spectra of ANSPI, PNSPI, ASPINC, PSPINC, TAPC(30 nm)/PSPINC and TAPC(50 nm)/PSPINC [Inset CIE image].

To evaluate EL performances of ANSPI, PNSPI, ASPINC and PSPINC we have fabricated non-doped devices with configuration of ITO/NPB(10 nm)/ANSPI or PNSPI or ASPINC or PSPINC (40 nm)**/**TPBi (15 nm)**/**LiF (1 nm)**/**Al (100 nm): ITO used as anode, 4,4′ -bis[N-(1-naphthyl)-N-phenyl-l-amino]biphenyl (NPB) used as hole transporting layer (HTL) and 1,3,5-tris(1-phenyl-1H-benzimidazol-2-yl)benzene (TPBi) used as electron transporting layer (ETL). The HOMO/LUMO energies of the materials are shown in Fig. 5. The operating voltage of pyrene compounds (PNSPI and PSPINC) based devices is lower than that of anthracene (ANSPI and ASPINC) devices which may be ascribed to better carrier transporting ability of PNSPI and PSPINC. The cyano pyrenyl emitter PNSPI based blue device (470 nm) shows high efficiencies (*η*_*c*_-3.92 cd**/**A; *η*_*p*_-2.01 lm**/**W; *η*_*ex*_-2.02%: Fig. 6) at 3.50 V with CIE (0.15, 0.18) than cyano anthracenyl emitter ANSPI device (465 nm) (*η*_*c*_-2.58 cd**/**A; *η*_*p*_-1.26 lm**/**W; *η*_*ex*_*-*1.82%) at 3.61 V with CIE (0.16, 0.17). The PSPINC based blue device (476 nm) shows high efficiencies (*η*_*c*_-4.23 cd/A; *η*_*p*_-2.86 lm/W; *η*_*ex*_-3.48%: Fig. 6) at 3.10 V with CIE (0.16, 0.17) than ASPINC device (469 nm) (*η*_*c*_-3.26 cd**/**A; *η*_*p*_-2.03 lm**/**W; *η*_*ex*_*-*2.86%) at 3.20 V with CIE (0.15, 0.14).

Among the pyrene compounds PNSPI and PSPINC and anthracene compounds ANSPI and ASPINC, PSPINC and ASPINC exhibit maximum efficiency due to better charge carrier transporting ability and the deeper emission which can be attributed to naphthonitrile group that induced effective molecular separation. Devices based on ANSPI, PNSPI, ASPINC and PSPINC show maximum brightness of 8356, 11823, 10123 and 12568 cd m^−2^ with blue emission at 465, 470, 469 and 476 nm, respectively (Fig. 4). The emission of anthracene compounds ANSPI and ASPINC are red- shifted around 19 nm compared to film emission which may be caused by the intermolecular interaction at the excited state. Since the compounds fabricated in device is in a thicker solid state, inevitably, the intermolecular interaction and the electrical field polarization in the excited state must be considered. However, the EL of pyrene compounds PNSPI and PSPINC are red-shifted only around 10 nm compared to PL in solid state: the electrical field polarization induced red shift of those compounds are attributed to separated HOMO**/**LUMO distribution. Meanwhile, the spatial steric configuration of these compounds also affects the electrical field polarization. The electroluminescence (EL) spectra of the devices show similar trends as PL in solid state because of EL peak with narrow FWHM of ANSPI, PNSPI, ASPINC and PSPINC are of 78, 65, 70 and 60 nm, respectively. The EL spectra of pyrene devices (PNSPI and PSPINC) are red shifted compared to anthracene devices (ANSPI and ASPINC) which can be ascribed to hypochromic shift of EL spectra and broader FWHM of ANSPI and ASPINC. Inspired by the efficient performance of PSPINC and its weak electrical field polarization effect, the blue electroluminescent device based on PSPINC was further optimized with the device configuration: The MoO_3_ used as hole injection layer, 1,1-bis[4-[N,N′-di(p-tolyl)amino]phenyl] cyclohexane (TAPC) was used as hole transporting layer (HTL) and TPBi functioned as electron transporting and hole blocking layer (Fig. 5; Table 2). The turn on voltage of the devices are in coincides with thickness of devices: device with TAPC (50 nm) show blue emission (428 nm) with maximum efficiencies *η*_*c*_-4.89 cd**/**A; *η*_*p*_-2.98 lm**/**W; *η*_*ex*_-3.93%, at 3.05 V than TAPC (30 nm) device (415 nm) (*η*_*c*_-4.68 cd**/**A; *η*_*p*_-2.90 lm**/**W; *η*_*ex*_*-*3.51%) at 2.96 V, however, the device based on thinner TAPC (30 nm) shows higher color purity [CIE (0.15, 0.17] than device with TAPC (50 nm) CIE (0.15, 0.18) which can be induced by the movement of exciton formation area.

**Table 2:** Device efficiencies of ANSPI, PNSPI, ASPINC and PSPINC.

Emitters	V (V)	L (cd/m^2^)	*η*_*ex*_ (%)	*η*_*c*_ (cd A^−1^)	*η*_*p*_ (lmW^−1^)	CIE (x, y)	EL (nm)
ANSPI (40 nm)	3.61	8356	1.82	2.58	1.26	0.16,0.17	465
PNSPI (40 nm)	3.50	11823	2.02	3.92	2.01	0.15,0.18	470
ASPINC (40 nm)	3.20	10123	2.86	3.26	2.03	0.15,0.14	469
PSPINC (40 nm)	3.10	12568	3.48	4.23	2.86	0.16,0.17	476
TAPC(30 nm)/PSPINC	2.96	10946	3.51	4.68	2.90	0.15,0.17	415
TAPC(50 nm)/PSPINC	3.05	13648	3.93	4.89	2.98	0.15,0.18	428
An(PPI)_2_:ANSPI	3.20	23098	5.86	10.68	5.46	0.15,0.18	459
An(PPI)_2_:PNSPI	3.10	10561	3.05	8.35	3.16	0.15,0.18	459
An(PPI)_2_:ASPINC	3.15	23986	6.79	12.13	5.98	0.15,0.17	458
An(PPI)_2_:PSPINC	2.95	11231	5.01	9.19	3.95	0.15,0.18	459
Ir(ppy)_3_:ASPINC	3.32	61581	17.0	53.4	50.6	0.31,0.60	501
(bt)_2_Ir(dipba):ASPINC	3.20	13456	14.5	16.5	15.2	0.63,0.36	610

### Characterization of doped fluorescent/phosphorescent OLEDs

**Figure 7:**
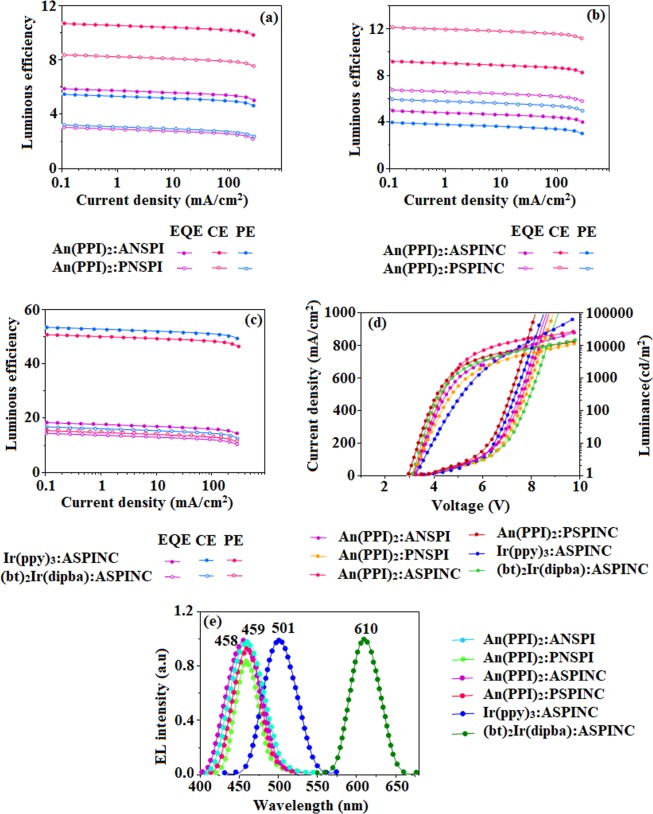
Device efficiencies of doped blue device: Luminous efficiency [CE (cd/m^2^), PE (lm/W)& EQE (%)] -Current density (**a**–**c**); Luminance –Voltage (**d**,**e**) EL spectra of An(PPI)_2_:ANSPI, An(PPI)_2_:PNSPI, An(PPI)_2_:ASPINC, An(PPI)_2_:PSPINC, Ir(ppy)_3_:ASPINC and (bt)_2_Ir(dipba):ASPINC.

The bis-4-(1,2-diphenyl-9,10-phenanthroimidazolyl) substituted anthracene (An(PPI)_2_, (fac-tris(2-phenylpyridine)iridium) (Ir(ppy)_3_)^79^, and bis-2-benzothiozolatophenyl iridium (III)- N, N’ -diisopropylbenzamidinate (bt)_2_Ir(dipba)^80^ are employed as blue fluorescent, green and red phosphorescent dopant. A large overlap (Fig. 4) between the absorption of dopant with emission of ASPINC results energy transfer (ET) to dopants. Since triplet energy (E_T_~2.60 eV) of these materials is lower than FIrpic~2.65 eV the incomplete host → dopant energy transfer leads to lower the efficiencies. ASPINC (E_T_) > FIrpic (E_T_), green and red emitters show triplet–triplet (Dexter) ET is possible along singlet–singlet (Forster) ET when doping green/red emitters into host ASPINC. Emission of 5 wt% of RGB dopants:host ASPINC film confirmed efficient energy transfer and λ_max_ of film is same with λ_max_ dopants λ_max_, however ASPINC λ_emi_ was not obtained. The single-exponential decay (2.7 ns, 1.01 ns and 0.83 ns) supports complete ET from ASPINC host → dopants show high-performance OLED devices. The slightly red-shifted emission from doped film to neat film of corresponding dopants should be related to the tighter packing between the dopant molecules in the neat film. The above studies show that ANSPI, PNSPI, ASPINC and PSPINC could be good host materials for both fluorescent and phosphorescent OLEDs. The efficient performance of the non-doped blue OLEDs prompted us to explore the possibility of using these blue emissive materials as host in host**/**dopant hybrid devices. To evaluate its practical utility, a series of blue fluorescent OLEDs with simple configuration of ITO**/**NPB (10 nm)/ANSPI or PNSPI or ASPINC or PSPINC (40 nm): 5% An(PPI)_2_/TPBi (15 nm)**/**LiF (1 nm)/Al (100 nm) were fabricated and the energy diagram of these materials used in the EL devices is shown in Fig. 5. NPB was used as the hole transporting material (HTL) and TPBi was used as the electron transport/hole-blocking layer (ETL/HBL) (Fig. 5). Similar with non-doped device, the operating voltage of pyrene compounds PNSPI and PSPINC based devices is lower than anthracene compounds ANSPI and ASPINC based devices which may be attributed to better charge carrier transporting ability of PNSPI and PSPINC. Among the pyrene compounds PNSPI and PSPINC, An(PPI)_2_:PSPINC based blue device (459 nm) shows high efficiencies (*η*_*c*_-9.19 cd/A; *η*_*p*_-3.95 lm**/**W; *η*_*ex*_-5.01%; L-11231 cd m^−2^) at 2.95 V with CIE (0.15, 0.18) than An(PPI)_2_:PNSPI device (459 nm) (*η*_*c*_-8.35 cd**/**A; *η*_*p*_-3.16 lm**/**W; *η*_*ex*_*-*3.05%; L-10561 cd m^−2^) at 3.10 V with CIE (0.15, 0.18) (Fig. 7). Considering the anthracene compounds (ANSPI and ASPINC), An(PPI)_2_:ASPINC shows high efficiencies (*η*_*c*_-12.13 cd/A; *η*_*p*_-5.98 lm/W; *η*_*ex*_-6.79%; L-23986 cd m^−2^; EL-458 nm) at 3.15 V with CIE (0.15, 0.17) than An(PPI)_2_: ANSPI device (459 nm) (*η*_*c*_-10.68 cd**/**A; *η*_*p*_-5.46 lm**/**W; *η*_*ex*_*-*5.86%; L-23098 cd m^−2^) at 3.20 V with CIE (0.15, 0.18) (Fig. 7). Among the doped blue fluorescent devices, An(PPI)_2_:ASPINC shows high efficiencies than An(PPI)_2_:PSPINC based device which is inconsistent with non-doped blue device performances.

The EL spectrum is consistent with the PL spectrum of An(PPI)_2_: ASPINC/ANSPI/PNSPI/PSPINC suggesting that the blue EL emission results from the intrinsic emission of An(PPI)_2_. However, the efficient overlap of emission spectra of ANSPI, PNSPI, ASPINC and PSPINC in film state with absorption spectra of the dopant (An(PPI)_2_) enhanced the efficiency (Fig. 4). The film of Ir(ppy)_3_ and (bt)_2_Ir(dipba) doped in ASPINC at 5 wt% concentration was used as emissive layer to fabricate the phosphorescent green and red devices, respectively. Similarly, green PhOLEDs with Ir(ppy)_3_:ASPINC (*η*_*c*_-50.6 cd/A; *η*_*p*_-53.4 lm/W; *η*_*ex*_-17.0%; L-61581 cd m^−2^; EL-501nm, CIE (0.31, 0.60)) at 3.32 V and red PHOLEDs with (bt)_2_Ir(dipba):ASPINC (*η*_*c*_-15.2 cd/A; *η*_*p*_-16.5 lm/W; *η*_*ex*_-14.5%; L-13456 cd m^−2^; EL-610 nm, CIE (0.63, 0.36)) at 3.20 V show higher efficiencies, such high and stable EL performance should be attributed to the balanced carrier injection/transport ability of ASPINC which result in a broad distribution of recombination region in the corresponding emissive layer. A low probability of triplet–triplet annihilation that causes an efficiency roll-off at high current density for the PhOLEDs. Charge confinement in the emissive layer is another key factor for high EL efficiency. There is a large energy barrier of ~0.6 eV prevents the hole leakage from ASPINC to TPBi together which prevents electron leakage from ASPINC to NPB. Therefore, holes and electrons can be effectively confined inside the ASPINC results in achieving high efficiency and low roll-off OLEDs. Besides, the E_T_ (~2.60 eV) of ASPINC is high enough than the phosphorescent dopants green [Ir(ppy)_3_, E_T_: ~2.4 eV)^81^ and red ((bt)_2_Ir(dipba), E_T_: ~2.1 eV]^82^ for working as a host, the energy loss during the host-to dopant energy transfer process can be reduced as far as possible^59^. The ASPINC was found to be the best host material for our devices. It is notable that the excellent performances of blue, green and red OLEDs were harvested from same device configuration by adopting same host material. The simple material system and easy fabrication process are of significance and importance for reducing the cost and enhancing the process stability in commercial mass production.

## Conclusion

We have reported the newborn deep-blue emitters ANSPI, PNSPI, ASPINC and PSPINC consist of anthracene, pyrene, styryl and phenanthro[9,10-d]imidazole fragments for molecular design strategy. These compounds exhibit excellent thermal properties with very high glass-transition temperature (*T*_*g*_) thus, stable thin film is formed on device fabrication. The pyrene containing PSPINC based non-doped blue device (476 nm) shows high efficiencies (*η*_*c*_-4.23 cd/A; *η*_*p*_-2.86 lm/W; *η*_*ex*_-3.48%: CIE (0.16, 0.17) at 3.10 V. Among the doped blue devices, An(PPI)_2_: ASPINC shows high efficiencies (*η*_*c*_-12.13 cd/A; *η*_*p*_-5.98 lm/W; *η*_*ex*_-6.79%; L-23986 cd m^−2^; EL-458 nm, CIE (0.15, 0.17)) at 3.15 V than An(PPI)_2_:PSPINC based device which is inconsistent with that of non-doped device performances. The energy transfer between the host ASPINC and dopant molecules improved the efficiency of the devices. Similarly, green and red PhOLEDs with Ir(ppy)_3_:ASPINC (*η*_*c*_-50.6 cd/A; *η*_*p*_-53.4 lm/W; *η*_*ex*_-17.0%; L-61581 cd m^−2^; EL-501nm, CIE (0.31, 0.60)) at 3.32 V and (bt)_2_Ir(dipba):ASPINC (*η*_*c*_-15.2 cd/A; *η*_*p*_-16.5 lm/W; *η*_*ex*_-14.5%; L-13456 cd m^−2^; EL-610 nm, CIE (0.63, 0.36)) at 3.20 V show higher efficiencies. The primary results suggest that an efficient energy transfer between fluorescence host and dopant are affected not only by Förster energy transfer mechanism but also the complicated interface effect among the hybrids.

### Experimental section

#### 1-(1-bromonaphthalen-4yl)-2-styryl-1H-phenanthro[9,10-d]imidazole (BNSPI)

A mixture of 9,10- phenanthrenequinone (2.08 g, 10 mmol), 4-bromonaphthalene-1-amine (0.93 g, 10 mmol), cinnamaldehyde (1.85 g, 10 mmol) and ammonium acetate (9.24 g, 120 mmol) in 50 ml acetic acid was refluxed for 3 days under nitrogen atmosphere. The mixture was cooled and poured into water. The isolated solid was washed with methanol and dried. Anal. Calcd. for C_33_H_21Br_N_2_: C, 75.45; H, 4.03; N, 5.33. Found: C, 75.39; H, 3.98; N, 5.25. ^1^H NMR - 400 MHz (CDCl_3_): δ 6.90 (d, *J* = 16.0 *Hz*, 1 H), 7.12–7.41 (m, 5 H), 7.41–7.55 (m, 4 H), 7.71–7.88 (m, 6 H), 8.13 (d, *J* = *16.2* *Hz*, 1 H), 8.24 (d, *J* = *8.8* *Hz*, 2 H), 8.95 (d, *J* = *7.8 Hz*, 2 H). ^13^C NMR - 100 MHz (CD^Cl^_3_): δ 112.83, 122.15, 122.47, 123.81, 126.48, 126.57, 128.06, 128.63, 128.75, 131.53, 132.72, 133.47, 135.67, 141. 53. MS: m/z. 524.18 [M^+^]. calcd. 524.09.

#### 4-(2-(4-bromostyryl)-1H-phenanthro[9,10-d]imidazol-1-yl)naphthalene-1-carbonitrile (BPINC)

A mixture of 9,10- phenanthrenequinone (2.08 g, 10 mmol), 4-aminonaphthalene-1-carbonitrile (0.93 g, 10 mmol), *(E)*-3-(4-bromophenyl)acrylaldehyde (1.85 g, 10 mmol) and ammonium acetate (9.24 g, 120 mmol) in 50 ml acetic acid was refluxed for 3 days under nitrogen atmosphere. The separated solid was washed with methanol and dried. Anal. Calcd. for C_34_H_20_BrN_3_: C, 74.19; H, 3.66; N, 7.63. Found: C, 74.05; H, 3.52; N, 7.45. ^1^H NMR - 400 MHz (CDCl_3_): δ 6.08 (d, *J* = *16.2* *Hz*, 1 H), 7.11 (d, *J* = ^*16.4*^ *Hz*, 1 H), 7.32–7.41 (m, 3 H), 7.52–7.61 (m, 2 H), 7.81–7.89 (m, 1 H), 8.12–8.20 (m, 5 H) 8.93 (d, *J* = *7.8 Hz*, 2 H). ^13^C NMR - 100 MHz (CD^Cl^_3_): δ 109.58, 112.13, 115.12, 121.73, 122.41, 123.75, 126.57, 126.62, 127.13, 128.65, 128.93, 131.52, 132.61, 133.89, 134.59, 136.08, 141.15. MS: m/z. 549.14 [M^+^]. calcd. 549.08.

#### 1-(1-(anthracen-10-yl)naphthalen-4-yl)-2-styryl-1H-phenanthro[9,10-d]imidazole (ANSPI)

A mixture of BNSPI (0.90 g, 2 mmol), anthracen-9-yl-boronic acid (0.49 g, 2.2 mmol), toluene (30 ml), 2 M K_2_CO_3_ (30 ml, 60 mmol), ethanol (15 ml) and tetrakis-(triphenylphosphine) palladium (0.12 g, 0.1 mmol) was refluxed under nitrogen for 36 h. The solution was extracted with dichloromethane and the organic layer was concentrated, purified by column chromatography and dried. Anal. Calcd. for C_47_H_30_N_2_: C, 90.65; H, 4.86; N, 4.50. Found: C, 90.56; H, 4.81; N, 4.42. ^1^H NMR - 400 MHz (CDCl_3_): δ 6.21 (d, *J* = *16.4* *Hz*, 1 H), 7.12–7.45 (m, 3 H), 7.51 (d, *J* = *16.8 Hz*, 1 H), 7.62–7.68 (m, 7 H), 7.82–7.89 (m, 4 H), 8.14 (d, *J* = *8.8 Hz*, 2 H) 8.95 (d, *J* = *8.2 Hz*, 2 H). ^13^C NMR (100 MHz, CD^Cl^_3_): δ 112.83, 122.32, 124.63, 126.11, 126.45, 126.97, 127.56, 127.83, 128.05, 128.53, 128.72, 129.27, 130.25, 131.24, 133.21, 134.79, 135.27, 136.18, 138.09, 141.05. MALDI-TOF MS: m/z 622.18, [M^+^]; calcd: 622. 24.

#### 1-(1-(pyren-1-yl)naphthalen-4-yl)-2-styryl-1H-phenanthro[9,10-d]imidazole (PNSPI)

A mixture of BNSPI (0.90 g, 2 mmol), pyren-4-yl-4-boronic acid (0.49 g, 2.2 mmol), toluene (30 ml), 2 M K_2_CO_3_ (30 ml, 60 mmol), ethanol (15 ml) and tetrakis-(triphenylphosphine) palladium (0.12 g, 0.1 mmol) was refluxed under nitrogen for 36 h. The solution was extracted with dichloromethane and the isolated solid was purified by column chromatography and dried. Anal. Calcd. for C_49_H_30_N_2_: C, 90.99; H, 4.68; N, 4.33. Found: C, 90.84 H, 4.51; N, 4.22. ^1^H NMR - 400 MHz (CDCl_3_): δ 6.15 (d, *J* = *16.8* *Hz*, 1 H), 7.14–7.46 (m, 8 H), 7.51 (d, *J* = *16.2* *Hz*, 1 H), 7.75–7.78 (m, 7 H), 7.81–7.88 (m, 6 H), 8.05–8.14 (m, 5 H) 8.91 (d, *J* = *8.4 Hz*, 2 H). ^13^C NMR - 100 MHz (CD^Cl^_3_): δ 112.81, 120.92, 122.43, 125.21, 125.45, 126.37, 126.56, 127.63, 128.01, 128.31, 131.12, 131.24, 132.41, 133.37, 133.91, 135.19, 136.09, 141.15. MALDI-TOF MS: m/z 646.31, [M^+^]; calcd: 646. 24.

#### 4-(2-(4-(anthracen-9-yl) styryl)-1H-phenanthro [9,10-d] imidazol-1-yl) naphthalene-1-carbonitrile (ASPINC)

The compound ASPINC was prepared using the methodology similar to that of ANSPI by replacing BNSPI with BPINC. Anal. Calcd. for C_48_H_29_N_3_: C, 89.00; H, 4.51; N, 6.49. Found: C, 88.75; H, 4.42; N, 6.35. ^1^H NMR - 400 MHz (CDCl3): δ 6.08 (d, *J* = *16.4* *Hz*, 1 H), 7.32–7.43 (m, 9 H), 7.54–7.66 (m, 7 H), 7.78 (d, *J* = *16.2* *Hz*, 1 H), 7.80–7.87 (m, 6 H), 8.12–8.20 (m, 3 H) 8.94 (d, *J* = *7.8 Hz*, 2 H). ^13^C NMR - 100 MHz (CD^Cl^_3_): δ 109.84, 112.53, 115.62, 121.73, 122.41, 123.75, 126.37, 126.66, 127.43, 127.65, 127.83, 129.52, 130.47, 131.67, 132.75, 133.54, 134.21, 134.81, 135.79, 138.19, 141.45. MALDI-TOF MS: m/z 647.16, [M^+^]; calcd: 647. 24.

#### 4-(2-(4-(pyren-1-yl)styryl)-1H-phenanthro[9,10-d] imidazol-1-yl)naphthalene-1-carbonitrile (PSPINC)

The compound PSPINC was synthesized using the methodology similar to that of PNSPI by replacing BNSPI with BPINC. Anal. Calcd. for C_50_H_29_N_3_: C, 89.39; H, 4.35; N, 6.25. Found: C, 89.32; H, 4.22; N, 6.14. ^1^H NMR - 400 MHz (CDCl_3_): δ 6.12 (d, *J* = *16.2 Hz*, 1 H), 7.36–7.45 (m, 5 H), 7.51–7.64 (m, 2 H), 7.81 (d, *J* = *16.4 Hz*, 1 H), 7.84–7.91 (m, 12 H), 8.04–8.21 (m, 6 H), 8.91 (d, *J* = *8.8* *Hz*, 2 H). ^13^C NMR - 100 MHz (CDCl_3_): δ 109.58, 112.03, 115.82, 120.93, 121.81, 122.45, 123.87, 125.36, 125.43, 126.35, 126.53, 126.82, 127.57, 127.67, 127.78, 127.94, 131.51, 132.31, 132.79, 133.49, 134.07, 135.78, 141.45. MALDI-TOF MS: m/z 671.18, [M^+^]; calcd: 671. 24.

### Measurements

NMR measurements were taken on Bruker spectrometer (400 MHz) and Agilent LCMS was employed to confirm mass of the emitters. The UV-optical spectra were measured on Lambda 35 PerkinElmer (solution)/RSA-PE-20integrated sphere (film) instrument. The emission spectra were analyzed with PerkinElmer LS55 fluorescence spectrometer measurements. The absolute quantum yield was measured with fluorescence spectrometer Model-F7100 with integrating sphere. The decomposition (*T*_*d*_) and glass transition (*T*_*g*_) temperature was measured with Perkin Elmer thermal analysis system (10 °C min^−1^; N_2_ flow rate − 100 ml min^−1^) and NETZSCH (DSC-204) (10 °C min^−1^; N_2_ atmosphere), respectively. Fluorescence lifetime was determined on Horiba Fluorocube-01-NL lifetime system. Oxidation potential of emissive materials were measured from potentiostat electrochemical analyzer (CHI 630 A). Ferrocene (HOMO−4.80 eV) was used as internal standard and 0.1 M tetrabutylammoniumperchlorate in CH_2_Cl_2_ as supporting electrolyte. The frontier energies were calculated by *E*_*HOMO*_ = −*(E*_*ox*_ + *4.8 eV*) and *E*_*LUMO*_ = *(E*_*red*_ − *4.8 eV)*, respectively.

### Computational details

The optimized geometry, HOMO and LUMO contour map were studied with Guassian-09 package [DFT/B3LYP/6–31 G (d, p)]^83^.

### Device fabrication and measurement

The ITO glass (resistance 20 Ω/sq) was cleaned with acetone, deionized water followed by isopropanol and dried at 120 °C in an oven and treated with UV-zone for 20 min and transferred into a deposition system. The devices were fabricated by multiple source organic molecular beam deposition method in a vacuum at a pressure of 4 × 10^−5^ mbar with evaporation rate of 1–2 Å/s for organic materials and LiF cathode of Al were deposited with the rates of 0.1 and 10 Å s^−1^, respectively. The thickness of each deposition layer was monitored using quartz crystal thickness monitor. The EL spectra and CIE coordinates were recorded with USB-650-VIS-NIR spectrometer (Ocean opitics, Inc., USA). The current density-voltage-luminance characteristics were studied by using computer-controlled source meter (Keithley 2450) equipped with light intensity meter LS-110 under ambient atmosphere without encapsulation. The external quantum efficiency was calculated from luminance, current density and EL spectrum assuming Lambertian distribution.

## Data Availability

The authors declare that data in our manuscript are available.
